# Congenital Hepatic Fibrosis Presenting With Pancytopenia

**DOI:** 10.1097/PG9.0000000000000043

**Published:** 2021-01-13

**Authors:** Vikas M. Mankala, Jessica L. Davis, Chirag V. Patel, Henry C. Lin

**Affiliations:** From the * Division of Pediatric Gastroenterology, Doernbecher Children’s Hospital, Portland, OR; † Department of Pediatrics, Oregon Health & Science University, Portland, OR; ‡ Department of Pathology, Oregon Health & Science University, Portland, OR; § Department of Diagnostic Radiology, Oregon Health and Science University, Portland, OR.

## INTRODUCTION

Congenital hepatic fibrosis (CHF) is a rare inherited fibrocystic disease of the liver characterized by ductal plate malformation, which is a developmental abnormality of the biliary system. CHF can be associated with ciliopathies, which have varying degrees of both liver and renal involvement. Cystic kidney diseases including autosomal polycystic kidney disease are the most common renal manifestation associated with CHF. Other common hepatic manifestations include secondary biliary strictures and periportal fibrosis, which can lead to portal hypertension and its associated sequelae. Common sequela of portal hypertension includes hypersplenism, esophageal varices, and ascites (1,2). As the spleen acts as a reservoir for red blood cells, platelets, and lymphocytes, cell line deficiencies such as anemia and thrombocytopenia can be seen in CHF. However, pancytopenia, while seen in hypersplenism, is an unusual presentation in children with CHF. We present a case of a boy with CHF who presented with hepatosplenomegaly and pancytopenia.

## CASE DESCRIPTION

**FIGURE 1.**
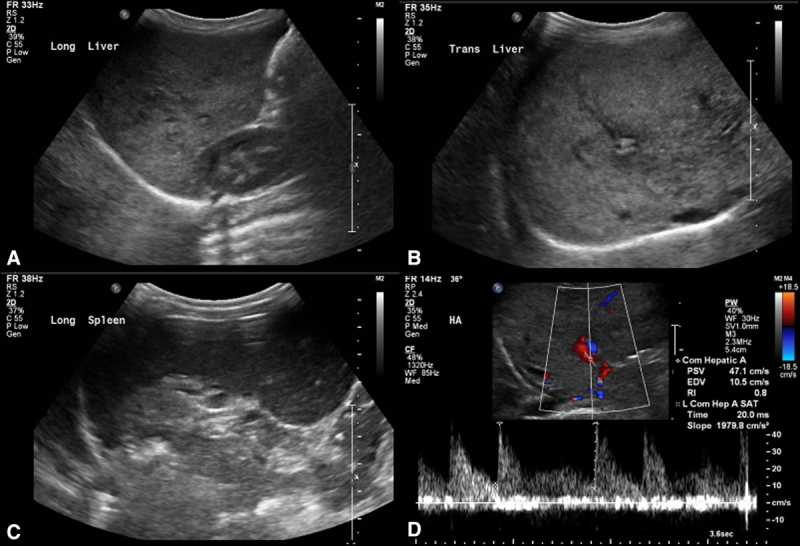
Ultrasound liver and spleen findings. Liver parenchymal echotexture is heterogeneous and hyperechoic (A and B). Spleen is enlarged (C). Hepatic artery demonstrates elevated resistive index at 0.8 (D).

A previously healthy 6-month-old male was noted to have mild splenomegaly on exam at his well-child check that was confirmed by ultrasound. Repeat ultrasound at 12 months of age showed hepatosplenomegaly with heterogeneous liver parenchyma and mildly elevated arterial resistive index (Fig. 1). Complete blood count was notable for pancytopenia with a white blood cell count of 3.91 K/cu mm, absolute lymphocyte count of 1820, hemoglobin of 9.7 g/dL, and platelets of 70,000.

He was admitted for further workup of pancytopenia, splenomegaly, and fever, with concern for possible leukemia or bone marrow infiltrative process, versus viral suppression. Blood cultures and infectious workup were negative including negative viral polymerase chain reaction testing for enterovirus, cytomegalovirus, adenovirus, and parvovirus. A bone marrow biopsy showed mildly hypocellular marrow (80%) with trilineage hematopoiesis and no evidence of acute leukemia or lymphoma. Workup for a metabolic etiology of hepatosplenomegaly including lysosomal storage diseases such as Gaucher disease, Niemann-Pick disease types A and B, Wolman disease, mucopolysaccharidosis I, GM1 gangliosidosis, and β-mannosidosis was negative. A week later, his pancytopenia spontaneously improved with only residual mild thrombocytopenia. Given this improvement, his pancytopenia was attributed to possible viral suppression.

**FIGURE 2.**
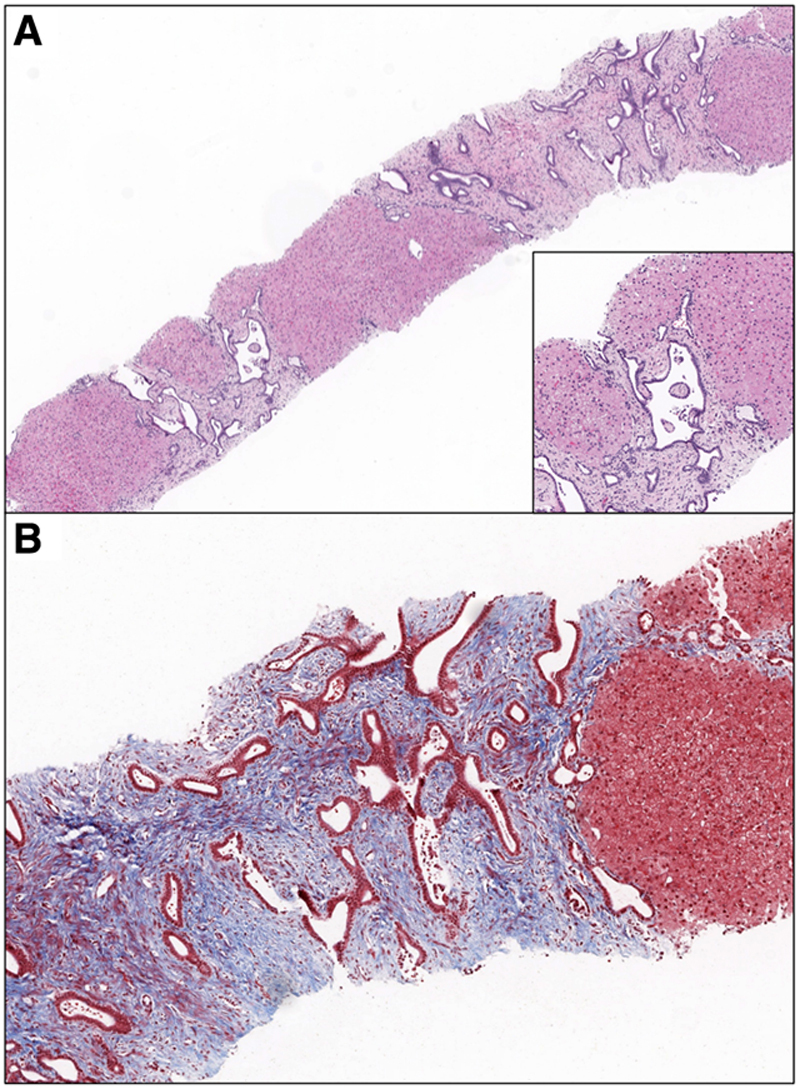
Congenital hepatic fibrosis. A, Bridging fibrosis containing multiple irregularly shaped, branching, and torturous duct structures (H&E ×40). Inset, Portal tract with irregular, dilated to saccular biliary structures consistent with duct plate malformation (H&E ×200). B, Degree of fibrosis highlighted by trichrome stain with fibrosis in blue (×100). H&E = hematoxylin-eosin.

Over the next few months, he developed intermittent pancytopenia with neutropenia followed by normalization of his cell counts. At 15 months of age, given the persistent hepatosplenomegaly in the setting of normal liver enzymes aside from a mildly elevated aspartate transaminase, gastroenterology was consulted for additional diagnostic workup. He ultimately had a liver biopsy performed at 16 months of age. Histology showed bridging fibrosis containing numerous irregular shaped, tortuous, and occasional saccular shaped duct structures (Fig. 2). The features are that of ductal plate malformation with CHF.

**FIGURE 3.**
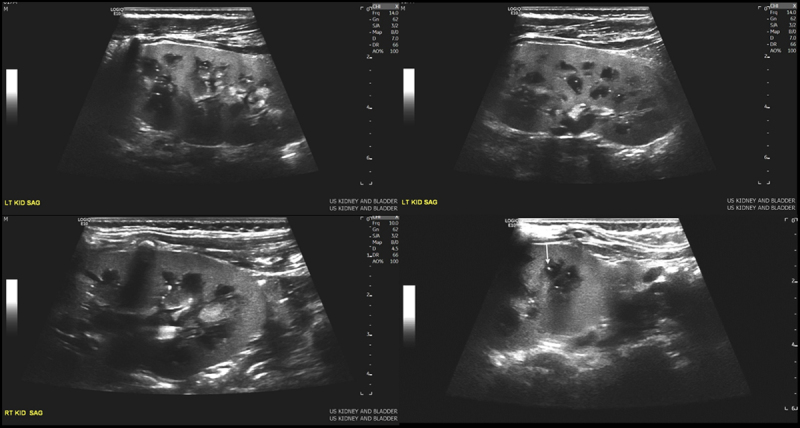
Hyperechogenic renal cortex and punctuate echogenic foci in the medullary pyramids.

With his diagnosis of CHF, he was referred to Nephrology. Renal ultrasound showed a hyperechogenic renal cortex, and while no macrocysts were identified in the kidneys, punctate echogenic foci in the medullary pyramids were noted, which can be seen in the setting of polycystic kidney disease (Fig. 3). Genetic sequencing of *PKHD1* gene identified 2 heterozygous mutations in *PKHD1*. He is heterozygous for a sequence variant c.930del, which is predicted to result in a frameshift and premature protein termination. He is also heterozygous for a sequence variant c.10319T>A, which is a variant of uncertain significance. His splenomegaly has been stable, and he has no other overt sequelae of portal hypertension. Liver function tests have been stable on routine monitoring with his most recent aspartate transaminase 71, alanine transaminase 30, alkaline phosphatase 146, total bilirubin 0.8, and direct bilirubin 0.3. He continues to have intermittent pancytopenia with his most recent white blood cell 2.48 with absolute neutrophil count of 940, hemoglobin 10.2, and platelets of 56,000.

## DISCUSSION

CHF is a rare disease of the hepatobiliary system characterized by ductal plate malformation (1). CHF can occur in isolation or in association with other ciliopathies, most commonly with autosomal recessive polycystic kidney disease. Other diseases in which CHF can be associated with include Bardet-Biedl syndrome, Joubert syndrome, nephronophthisis, congenital defect of glycosylation type 1b, Meckel-Gruber syndrome, orofacial digital syndrome, and autosomal dominant polycystic kidney disease (3). The clinical presentation of CHF can vary from primarily hepatic symptoms such as abdominal distension, hepatosplenomegaly, jaundice, and failure to thrive to hematologic signs such as thrombocytopenia or anemia. Other symptoms of CHF are a consequence of complications from portal hypertension such as ascites or hematemesis. Diagnosis of CHF is histologic with liver biopsy findings of cystic dilation of the intrahepatic bile ducts, abnormal intrahepatic portal vein branching, and portal fibrosis.

Here, we present the unique case of a patient with CHF who presented with hepatosplenomegaly and pancytopenia. The differential of pancytopenia and hepatosplenomegaly in an infant includes oncologic, infectious, and metabolic etiologies. Oncologic etiologies include leukemia, lymphoma, neuroblastoma, and infiltrative processes such as histiocytosis (4,5). Infectious etiologies include cytomegalovirus and Epstein-Barr virus, disseminated tuberculosis, coccidioidomycosis, blastomycosis, and toxoplasmosis (4,5). Metabolic etiologies include lysosomal storages disorders such as Gaucher disease, Niemann-Pick disease, Wolman disease, mucopolysaccharidosis I, GM1 gangliosidosis, β-mannosidosis, as well as select glycogen storage disorders such as Hurler’s (4,5). An underlying hepatic etiology should be considered as well, as hepatosplenomegaly can be seen in portal hypertension. Given the spleen’s role in the storage of red blood cells and platelets, splenic sequestration can contribute to pancytopenia, but typically, thrombocytopenia is the predominant hematologic abnormality.

This case additionally highlights the rarity of thrombocytopenia occurring in the pediatric population as a result of increased platelet sequestration from liver disease and portal hypertension. Thrombocytopenia as a whole is a very common problem in pediatrics, and can result from increased destruction, or decreased production of platelets. Immune-mediated destruction constitutes the majority of cases of thrombocytopenia due to increased platelet destruction, while liver-related sequela such as hypersplenism constitutes a small minority. One study on the etiology of thrombocytopenia in children found that 32% presented with thrombocytopenia due to immune thrombocytopenic purpura, while only 3% presented with thrombocytopenia due to hypersplenism (6). Thus, when thrombocytopenia persists, rare etiologies must be considered such as liver disease.

The diagnostic workup for hepatosplenomegaly and pancytopenia should be targeted to the suspected underlying etiology. While various conditions can present with hepatosplenomegaly and pancytopenia, few conditions also present with fever, which usually leads to a focus on oncologic or infectious etiologies. As oncologic or infectious etiologies can require a timely diagnosis to initiate appropriate treatment, initial evaluation may focus on these etiologies including targeted screening for infectious diseases based on history and a bone marrow biopsy to assess for oncologic etiology as in our patient. As the initial workup for infectious and oncologic etiologies was negative, the diagnostic focus turned to an underlying hepatic etiology, and our patient ultimately had a liver biopsy leading to diagnosis.

In our review of the literature, there was 1 reported case of a child with autosomal recessive polycystic kidney disease and CHF who presented with pancytopenia (7) and another case report of a boy with CHF and pancytopenia; however, this patient did not have hepatosplenomegaly and had a normal liver on abdominal ultrasound (8). In addition, there have been some case reports of children with CHF who copresented with thrombocytopenia and neutropenia, but these patients did not have deficiencies in all 3 cell lines as in our patient (9). While there are many other more prominent causes to explain pancytopenia, CHF should be considered in the differential in a child with pancytopenia and hepatosplenomegaly.

## CONCLUSIONS

We report a unique case of a child with CHF who presented with hepatosplenomegaly, pancytopenia, and fever. The presentation of fever lead to the initial focus of oncologic or infectious etiologies, as these conditions often require timely diagnosis, but testing was unremarkable. He was ultimately diagnosed with CHF on liver biopsy. While CHF can be associated with deficiencies in 1 or 2 cell lines, pancytopenia can occasionally be seen. While there are many etiologies to explain pancytopenia, CHF should be considered in the differential in a child with pancytopenia and hepatosplenomegaly.
